# A Hierarchical Spatial-Temporal Embedding Method Based on Enhanced Trajectory Features for Ship Type Classification

**DOI:** 10.3390/s22030711

**Published:** 2022-01-18

**Authors:** Tao Sun, Yongjun Xu, Zhao Zhang, Lin Wu, Fei Wang

**Affiliations:** 1 Institute of Computing Technology, Chinese Academy of Sciences, Beijing 100190, China; suntao@ict.ac.cn (T.S.); xyj@ict.ac.cn (Y.X.); zhangzhao2021@ict.ac.cn (Z.Z.); wulin@ict.ac.cn (L.W.); 2 School of Computing Science and Technology, University of Chinese Academy of Sciences, Beijing 100039, China

**Keywords:** ship classification, spatial-temporal embedding, feature enhancement, deep learning, attention

## Abstract

Ship type classification is an essential task in maritime navigation domains, contributing to shipping monitoring, analysis, and forecasting. Presently, with the development of ship positioning and monitoring systems, many ship trajectory acquisitions make it possible to classify ships according to their movement pattern. Existing methods of ship classification based on trajectory include classical sequence analysis and deep learning methods. However, the real ship trajectories are unevenly distributed in geographical space, which leads to many problems in inferring the ship movement mode on the original ship trajectory. This paper proposes a hierarchical spatial-temporal embedding method based on enhanced trajectory features for ship type classification. We first preprocess the trajectory and combine the port information to transform the original ship trajectory into the moored records of ships, removing the unevenly distributed points in the trajectory data and enhancing key points’ semantic information. Then, we propose a Hierarchical Spatial-Temporal Embedding Method (Hi-STEM) for ship classification. Hi-STEM maps moored records in the original geographical space into the feature space and can efficiently find the classification plane in the feature space. Experiments are conducted on real-world datasets and compared with several existing methods. The result shows that our approach has high accuracy in ship classification on ship moored records. We make the source code and datasets publicly available.

## 1. Introduction

Ship type classification is an essential task in maritime navigation domains [1,2,3,4]. We can analyze ship movement characteristics better by ship types, which contributes to shipping monitoring, analysis, and forecasting. Ship types can be obtained by some approaches, such as LRF (Lloyd’s Register Foundation (https://www.lrfoundation.org.uk/, accessed on 25 November 2021)) or AIS (Automatic Identification System (https://en.wikipedia.org/wiki/Automatic_identification_system, accessed on 25 November 2021)). However, the information in AIS will face the following problem: The ship type contained in the AIS message may not be the real type of the corresponding ship. There are two reasons for this: (1) Ship type in the AIS message is filled in manually, and some crew members may fill it in incorrectly. (2) Some ships will deliberately conceal the true type when carrying out illegal activities, such as smuggling, illegal fishing, illegal operations, etc. As a result, inferring ship types by their trajectories is an effective way to deal with this problem. Presently, with the development of ship positioning and monitoring system, it is possible to classify ships according to the ship’s movement pattern, and researchers have focused on this [5,6,7,8,9].

There are many general trajectory classification methods that do not only focus on ship classification. Trajectory classification has been widely studied and applied [10,11]. We mainly introduce four kinds of trajectory classification methods. One is based on trajectory similarity measure methods such as *Euclidean Distance* [12], *Hausdorff Distance* [13], *Dyn amic Time Warping Distance (DTW)*, *Longest Common Subsequence (LCSS) Distance*, and *Fréchet Distance*. Another trajectory classification method is based on trajectory cluster analysis [14,15,16,17]. Existing sequence inference models can be also employed in trajectory classification, such as Dynamic Bayesian Network (DBN) [18], and Hidden Markov Model (HMM) [19] which incorporate the information from locations and the sequential patterns between adjacent locations [10]. In recent years, artificial intelligence has been widely used in various fields [20]. Refs. [21,22,23,24] employ deep recurrent neural network for trajectory classification. Ref. [24] combines feature selection and deep learning for trajectory classification. Ref. [25] establishes a deep learning-based trajectory classification method under trajectory data flow. However, there are not many methods for ship trajectory classification. Compared with general trajectory classification, the proprietary ship classification method takes into account the uncertain characteristics so as to improve the accuracy of ship classification.

The most critical challenge of ship classification based on trajectory lies in the sparsity of ship trajectories [26], which is caused by the ship trajectory data acquisition approaches. Existing data acquisition methods include terrestrial-based AIS (T-AIS) equipment (https://www.marinetraffic.com/blog/seven-things-know-ais/, accessed on 25 November 2021) and a few satellite-based AIS (S-AIS) (AIS was not anticipated to be detectable from space. Nevertheless, since 2005, various entities have been experimenting with detecting AIS transmissions using satellite-based receivers and, since 2008, companies such as exactEarth (https://www.exactearth.com/, accessed on 25 November 2021), ORBCOMM (https://www.orbcomm.com/, accessed on 25 November 2021), Spacequest (https://www.aac-clyde.space/who-we-are/our-companies/spacequest, accessed on 25 November 2021), Spire (https://spire.com/, accessed on 25 November 2021) and also government programs have deployed AIS receivers on satellites.) Because most ship trajectory data are collected by T-AIS, it will lead to dense and complete ship trajectories near the shore and sparse and missing ship trajectories at the far shore [27], which will cause the existing methods to misjudge the ship type based on the sparse trajectory.

This paper establishes a hierarchical spatial-temporal embedding method based on enhanced trajectory features for ship type classification. First, through preprocessing, we transform the original ship trajectory with uneven distribution into moored records, which reduces and enhances semantic information. Furthermore, we propose a ship moored records classification method called **Hi-STEM**, a Hierarchical Spatial-Temporal Embedding Method, which maps the moored records from the original space into feature space, with spatial and temporal information remaining. We employ semi-supervised learning to embed the ports of moored records, enhancing the semantic information of ports. Hi-STEM uses recurrent neural networks to capture the sequence information of moored records. The original trajectory’s spatial and temporal information is retained by using hierarchical embedding, and the same type of trajectories is clustered in the feature space.

Our contributions are as follows:

- We establish a framework for ship classification based on ship moored records. In this way, we introduce the port information, which increases the features of the trajectory and reduces the problem of uneven data distribution.
- We propose a spatial-temporal sequence classification method called Hi-STEM. Hi-STEM processes the temporal and spatial information simultaneously and uses the attention recurrent neural network to improve the classification accuracy of sequences.
- We verify the effectiveness and the robustness of our method on real-world datasets. The results show that our method can arrive over 80% accuracy on ship classification of four categories, beyond the naive deep neural network approach.
- We arrange the datasets for ship classification, which contains information such as ship type, the ship moored records, and port basic properties. Details of datasets and source code can be obtained at the website https://github.com/taos123/Ship_Classification_Moored (accessed on 25 November 2021).

The rest of the paper is assigned as follows: In Section 2, we introduce the materials and methods in this paper. In Section 3, we present the detail of the experiments and show the results. Moreover, we demonstrate trajectory visualization analysis. We conclude our work and give the future work in Section 4.

## 2. Materials and Methods

In this section, we will introduce the details of the hierarchical spatial-temporal embedding method based on enhanced trajectory features for ship type classification. Our method consists of two components: First, we will describe the datasets used in this paper and introduce how to preprocess origin ship trajectories to obtain ship moored records. Second, we will introduce a spatial-temporal sequence classification method, Hi-STEM, which infers the ship type according to the moored records considered both spatial and temporal information.

### 2.1. Data Preprocessing

Due to the limitation of trajectory acquisition devices, there are a lot of redundant points in the original trajectory. Some of these points are meaningful, such as a ship staying in a port. However, some of the points have low information. These points will cause too many points in the whole trajectory and bring a certain amount of noise. Therefore, before the work of trajectory classification, we extract the critical position points in the trajectory. We believe that the behavior of ships calling at the port has more obvious semantic characteristics than sparse navigation. Therefore, we first extract the behavior of ships calling at the port from the ship trajectory, which we call ship moored records. To obtain the moored records of ships, we used two data sets. We employ real-world ship movement data collected from AIS (Automatic Identification System), which captures the movement of ships worldwide. There are many critical problems in vessel movement trajectory data, such as forging identity, cheating GPS location, going into the dark, etc. We clean the original data to solve the format problems and then extract the locations of the trajectories follow as [26].

**Figure 1:**
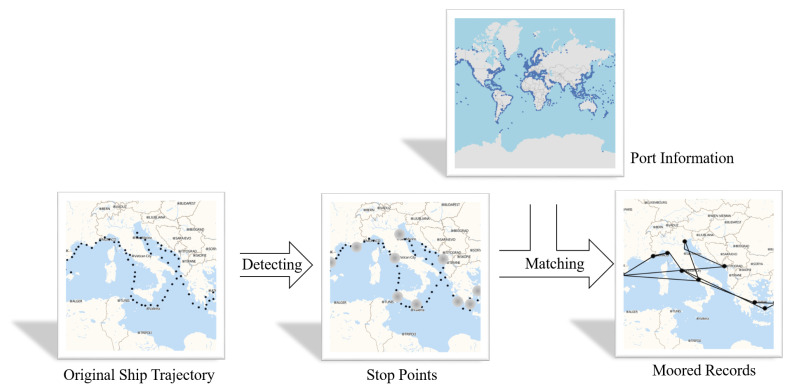
The demonstration of trajectory preprocess. The inputs include ship trajectories and port information. After the stop points detection and port matching, the output are ship moored records.

On the other hand, we also use the information of all ports in the world, which is crawled from World Port Index (https://msi.nga.mil/Publications/WPI, accessed on 25 November 2021), including the location, name, country, and other basic information of the port. The port data includes 3665 ports information. The calculation process of ship moored record includes two main processes, which is shown in Figure 1.

**Figure 2:**
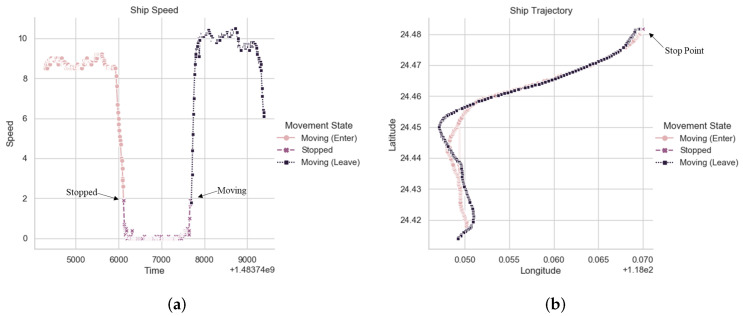
An example of ship point detection. Different colors lines correspond to different navigation states in the figure. (**a**) Ship Speed Variation Curve; (**b**) Ship Movement Trajectory.

Stop points detection is a crucial step of moored records calculating [28]. We calculate the stop point according to the operation state of the ship. We illustrate the calculation method with a specific example which is shown in Figure 2. First, we calculate the running speed of the ship. When the running speed of the ship is lower than a certain threshold for a certain period, it is considered that there is a stopping state.

Finally, we match the stop point with the ports to obtain the final moored records. For each stop point, we compare the point with all port locations and set a threshold. In practice, we set the threshold as 10 nautical miles by experience. If the distance between the stopping point and the port is less than the threshold, the ship is considered to be mooring at the port. If the distance between the stopping point and multiple ports at the same time is less than the threshold, we choose the port with the smallest distance as the stopping position.

By trajectory preprocess, the form of data has changed. We give a formal description. x,y,t→s,h,t. Where *s* denotes the port that the ship moored. *h* denotes the behavior of moving ships in *s*, which is defined as stay behavior in this paper. *t* is the occurrence time of stay behavior.

### 2.2. Hierarchical Spatial-Temporal Embedding Method for Ship Classification

**Figure 3:**
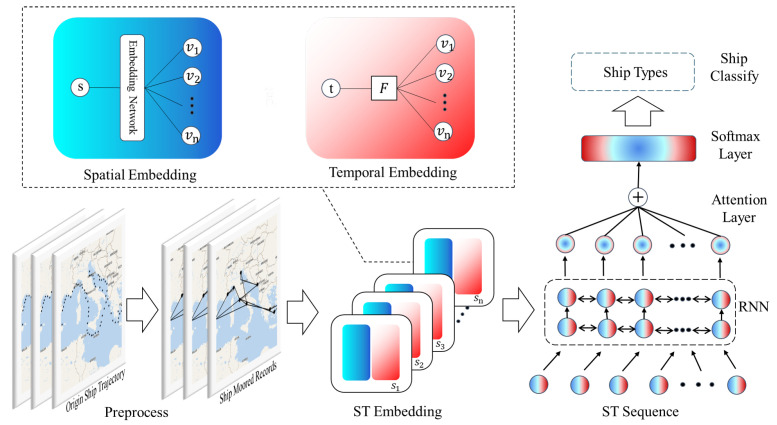
Overall structure of hierarchical spatial-temporal embedding method based on enhanced trajectory features for ship classification.

In this section, we introduce the hierarchical spatial-temporal embedding method for ship classification. For each moored record, we need to consider both spatial-temporal information and sequence information. To deal with it, we propose a spatial embedding method to enhance spatial semantic information through an unsupervised learning method, which will map ports into vector space. At the same time, we also introduce how to use the temporal information of moored records by the temporal embedding function. An attention recurrent neural network is employed for sequence classification. We will describe these steps in detail below. The overall structure of our method is shown in Figure 3.

#### 2.2.1. Spatial-Temporal Embedding

The ports that moored records contained represent the movement preference of the moving ships, which is an essential factor in differentiating the ship types. In ship navigation, the port is functional. Therefore, we hope to obtain the semantic information of the port to enhance the trajectory features. As mentioned in the domains of the natural language process, we can employ semi-supervised learning on the large-scale corpus to obtain word vectors that project words of the same meaning to similar vectors [29,30]. In the trajectory processing method, we also use many trajectory data to obtain the port embedding vector. For example, cargo transportation generally has the trajectory of movement from the port of cargo export, through the port of cargo import, and finally back to the port of cargo export; the passenger ship from the passenger terminal through the tourist resort and finally back to the passenger terminal. Of course, the actual situation of the moored records will be more complex than the above examples. Nevertheless, a large number of the moored records contain position information we can learn from. So we design an unsupervised learning spatial information representation method, which employs the structural information in moored records to learn the similar relationship between ports.

We construct the set of ports represented by a one-hot representation approach. In the preprocessing, we have used set $S=\{s_{1},s_{2},\cdots ,s_{l}\}$ to represent all the ports, where *l* is the size of the port set. An L-dimensional vector is used to represent all $s_{i}$ such as $\left[0,0,\cdots 1\cdots 0,0\right]$, where one is located on *i*th element and the rest are zero. Let $l\left(s_{i}\right)$ denotes the one-hot representation of $s_{i}$. For each port, the first and the last points adjacent to the original moored records are selected to form a corpus pair, where are the selected center port and the adjacent port sets. We extract from the moored records $T=(s_{1},s_{2},\cdots ,s_{n})$ from the moored record sets T as a location and its context. The training sample can be extracted as Equation (1).

(1) $$s_{i}∼<s_{i-1},s_{i+1}>$$

In the above equation, $s_{i}$ is the port of moored records *T* and $<s_{i-1},s_{i+1}>$ are the context of $s_{i}$ with context length 1. Let v(s) denotes the ports vector and F() denotes a measurement equation. A learning network is built to learn the information coding of trajectory points. The neural network is used as the learning network of trajectory point information coding. A port and its context, which are also called corpus, are the input and the output of the learning networks for training. Both the input and output use the unique hot coding of ports. We can train a simple neural network parameter *W* with training data sample $s_{i},s_{i-1},s_{i+1}$. The parameter v(s) of the neural network is the embedded port vector we need. Finally, we extract the last layer vector of the learning network as the information coding of trajectory points and use the representation. The whole process of acquiring position vector is the optimal solution of Equation (2).

(2) $$\begin{array}{cc} p\left(v\left(s_{i}\right)|C\left(s_{i},s\right)\right)\; & =\prod_{l^{\prime }\in C\left(s_{i},s\right)}s\left(v\left(s_{i}\right)|v\left(s^{\prime }\right)\right)\\ \; & =\prod_{s^{\prime }\in C\left(s_{i},s\right)}\frac{\exp\left\{v\left(s_{i}\right)\cdot v\left(s^{\prime }\right)\right\}}{\sum_{s^{\prime \prime }\in C\left(s_{i},s\right)}\exp\left\{v\left(s^{\prime \prime }\right)\cdot v\left(s^{\prime }\right)\right\}} \end{array}$$

where $v\left(s_{i}\right)$ represents the port vector representation and $C(s_{i},s)$ is the context of $s_{i}$.

The temporal information is also an important factor for inferring ship type with the moored records. Generally, the temporal information in the original trajectory data is usually represented by a positive integer, which represents the period from a fixed starting point to the time when the trajectory point is collected. However, this kind of time information cannot be directly processed. We formed a point for temporal information processing which processes the original temporal information into a representation that can extract temporal periodicity and is relatively homogeneous with spatial information. Let us use the following steps to first extract the year, month, day, hour, minute, and second information in the time information, as follows:

(3) $$t\to t_{y},t_{M},t_{d},t_{H},t_{m},t_{s}$$

Gaussian distribution is used to project time. Let us take component Y of time in the year as an example. There are two reasons that we project time: (1) due to all element values of the space embedding vector are in $\left[0,1\right]$, the corresponding element of time vector should also be in $\left[0,1\right]$. (2) The input of neural network should be continuous, not discrete. The treatment method is shown by the following formula.

(4) $$\begin{array}{cc} v_{t_{y}}\; & =G(t_{y})\\ \; & =\frac{1}{\sqrt{2\pi }}\exp\left(-\frac{{t_{y}}^{2}}{2}\right) \end{array}$$

In the same way, we can deal with the component of time in a month, day, hour, minute, and second. Finally, we splice the spatial embedding vector v(s) and the temporal embedding vector v(t) to form the vector represented by the trajectory point v(p).

#### 2.2.2. Spatial-Temporal Sequence Classification

At the same time, we need to consider the influence of moored records sequence information on ship classification results. Because the moored records data collected from the real world are affected by the movement of the objects and the adoption rate of the acquisition equipment, the trajectory data is often indefinite, so we need to consider the information of the port on each time node and judge the category of the ship by synthesizing this information. We combine recurrent neural networks model and moored records port to embed information in different periods of the records.

We take LSTM as the example in Hi-STEM. A common LSTM unit is composed of a cell, an input gate, an output gate and a forget gate. The cell remembers values over arbitrary time intervals and the three gates regulate the flow of information into and out of the cell. LSTMs were developed to deal with the exploding and vanishing gradient problems that can be encountered when training traditional RNNs. For the trajectory $T=\left\{p_{1},p_{2},\cdots ,p_{k}\right\}$, and let $h_{t-1}$ denote the last state, $h_{t}$ denotes the current state and $\tilde{h_{t}}$ denotes the candidate state. We can use the recursion formula as follows.

(5) $$\begin{array}{cc} f_{t} & =\sigma_{g}\left(W_{f}v\left(p_{t}\right)+U_{f}h_{t-1}+b_{f}\right)\\ i_{t} & =\sigma_{g}\left(W_{i}v\left(p_{t}\right)+U_{i}h_{t-1}+b_{i}\right)\\ o_{t} & =\sigma_{g}\left(W_{o}v\left(p_{t}\right)+U_{o}h_{t-1}+b_{o}\right)\\ c_{t} & =f_{t}\circ c_{t-1}+i_{t}\circ \sigma_{c}\left(W_{c}v\left(p_{t}\right)+U_{c}h_{t-1}+b_{c}\right)\\ h_{t} & =o_{t}\circ \sigma_{h}\left(c_{t}\right) \end{array}$$

In the training process, the initial values are $c_{0}=0$ and $h_{0}=0$ and the operator ∘ denotes the Hadamard product. The subscript *t* indexes the time step. $v\left(p_{t}\right)$ is input vector to the LSTM units. $f_{t}$ is the forget gate’s activation vector. $i_{t}$ is the update gate’s activation vector. $o_{t}$ is the output gate’s activation vector. $h_{t}$ is the hidden state vector also known as the output vector of LSTM units. $c_{t}$ is cell state vector. *W*, *U*, and *b* are weight matrices and bias vector parameters that need to be learned during training. $\sigma_{g}$ is a sigmoid function. $\sigma_{c}$ is a hyperbolic tangent. In this way, we can obtain the RNN output every time. GRU and Bi-LSTM are also employed in Hi-STEM.

The attention mechanism is introduced in Hi-STEM, which highlights the information of critical ports in the moored records when classifying the ships to improve the classification accuracy. When considering the influence of ports in different periods of a sequence on the results of ship classification, we will face the following problems: many different categories of moored records have a large number of coincidence sub-sequence, which leads to difficulty in distinguishing such moored records with repeated sub-sequence. In this regard, our strategy is to highlight the influence of different parts of moored records on the classification results in the process of classification to reduce the difficulty of the same moored records sequence on the classification results. To achieve those ideas, we introduce the attention mechanism in Hi-STEM. The core idea is to highlight the impact of the key port on the classification results in the process of trajectory classification to improve the accuracy of classification [31,32]. In this process, we need to consider how to determine the key ports in the moored records. In this regard, we introduce a corresponding weight factor to each position point in the sequence, where the weight factor is normalized, so that it is convenient to calculate the moored records affected by the weight factor. After obtaining the weight factor, the recalculated embedding vector can be expressed as Equation (6).

(6) $$c_{t}=\sum_{i=1}^{len(T)}a_{ts}h_{s}$$

Ports corresponding to each vector in $h_{s}$ trajectory points, $a_{ts}$ represents the weight factor. We also need to determine how to calculate the weight factor. Our method is to calculate the weight factor of each port and the correlation coefficient of the final classification result as Equation (7).

(7) $$a_{t}=f\left(c_{t},h_{t}\right)$$

(8) $$a_{ts}=\frac{\exp\left(score\left(h_{t},{\bar{h}}_{s}\right)\right)}{\sum_{s^{\prime }=1}^{S}\exp\left(score\left(h_{t},{\bar{h}}_{s^{\prime }}\right)\right)}$$

After normalizing the correlation coefficient calculated by the above formula, the weight factor needed in Equation (8) can be obtained.

After completing the process of hierarchical spatial-temporal embedding, we obtain a latent vector of trajectory, which is used for ship classification. The process of ship classification includes training and reasoning. Let us first describe the training process. We construct a multilayer perceptron, which consists of three layers of nodes: an input layer, a hidden layer, and an output layer, to map multiple input vectors to a single output vector. Except for the input nodes, each node is a neuron that uses a nonlinear activation function. It can distinguish data that is not linearly separable. One layer perceptron can be described by Equation (9) and it can be superimposed as MLP.

(9) z=tanh(W·x+b)

In the output layer, we use a softmax function. We use *z* to represent the input vector of the softmax function and *y* to represent the output vector of the softmax function, where $z_{i}$ is the *i*th element of the vector *z* and $y_{i}$ is the *i*th element of the vector *y*. For each element $z_{i}$ in the output layer, after softmax function processing, $y_{i}$ can be obtained as Equation (10).

(10) $$p\left(y_{i}\right)=\frac{e^{z_{i}}}{\sum_{j}e^{z_{j}}}$$

Moreover, we use part of the labeled moored records data as training data to input our method, and we use the method of random gradient descent to make the recognition model reach the optimal solution.

Then we employ the trained for ship classification reasoning. We use the original trajectory data as the input of the whole model. After preprocessing, layered embedding, and adjusting the weight of trajectory points, the position of the largest element in the output vector of MLP is the corresponding classification label. In this article, we classify ships into four categories, including cargo ship, fishing ship, oil ship and passenger ship. In the calculation process, we use a 1×4 vector to represent each different ship type. Specifically, $\left[1,0,0,0\right]$ denotes cargo ship, $\left[0,1,0,0\right]$ denotes fishing ship, $\left[0,0,1,0\right]$ denotes oil ship, and $\left[0,0,0,1\right]$ denotes passenger ship.

### 2.3. Parameter Setup

We set the iteration number of spatial embedding learning as 100. The size of the spatial embedding vector is 300. The initial learning rate of our method is 0.001. The number of the hidden layer and the attention layer of Hi-STEM is 300. We compare the performance among LSTM, GRU, and BRNN and use BRNN as the default recurrent module of our model. Other parameters can be looked up in the source code.

## 3. Results

In this section, we verify the effectiveness and robustness of ship classification method based on moored records. In addition, we visualize the embedded vectors and compare them with the moored records in geographical space to intuitively show that the hierarchical embedding method can effectively cluster ships of the same type in the embedding space. Before we show the results, we need to introduce the metric methods and ship classification baselines.

### 3.1. Datasets

**Figure 4:**
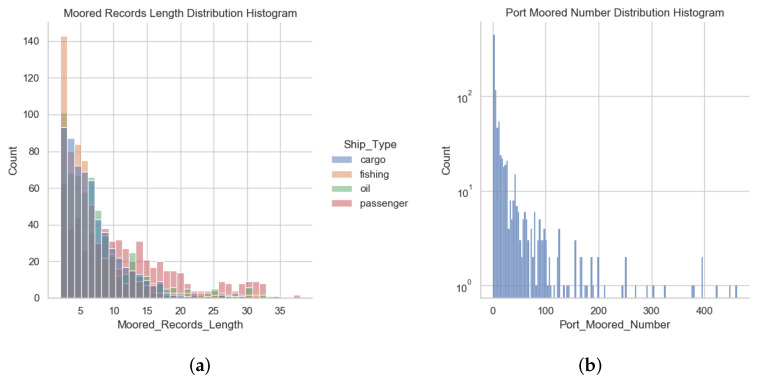
Ship moored records distribution histogram. From the perspective of ships and ports, we calculate the distribution of moored records, and counted ship moored record distribution according to ship types. (**a**) Moored Records Length Distribution Histogram; (**b**) Port Moored Number Distribution Histogram.

Our datasets consist of three parts. The main part records the ship’s moored record, including 20572 lines of data, and each line records the one moored record of a ship. The remaining two parts correspond to the basic information of the ship and the basic information of the port, respectively. The ship type, port location, and other basic information can be queried in the remaining two parts through the ship ID and port ID in the main part. We checked the ship types of each ship one by one to ensure that the ship types used for training and testing are correct. The detailed description of these datasets can be used at links https://github.com/taos123/Ship_Classification_Moored (accessed on 25 November 2021). The basic statistics of the datasets are shown in Table 1. The statistical distribution histogram of ship moored records and the statistical distribution histogram of port moored number are shown in Figure 4.

### 3.2. Evaluation Metrics

Because trajectory classification is a multi-classification task, we use the precision rate and recall rate to measure ship classification performance, the standard metric methods in information retrieval domains. Those metric methods are defined as follows.

$$precision=\frac{1}{N}\sum_{i=1}^{N}\frac{TP_{i}}{TP_{i}+FP_{i}}$$

$$recall=\frac{1}{N}\sum_{i=1}^{N}\frac{TP_{i}}{TP_{i}+FN_{i}}$$

In the above formula, $TP_{i}$, $FP_{i}$, and $FN_{i}$ are the number of true positive, false positive, false negative samples of category *i* in trajectory classification. *N* is the number of categories of trajectory datasets. F1 value is also employed to measure the model’s performance, which is defined as follows.

$$F1=2\times \frac{precision\times recall}{precision+recall}$$

### 3.3. Effectiveness Analysis

**Figure 5:**
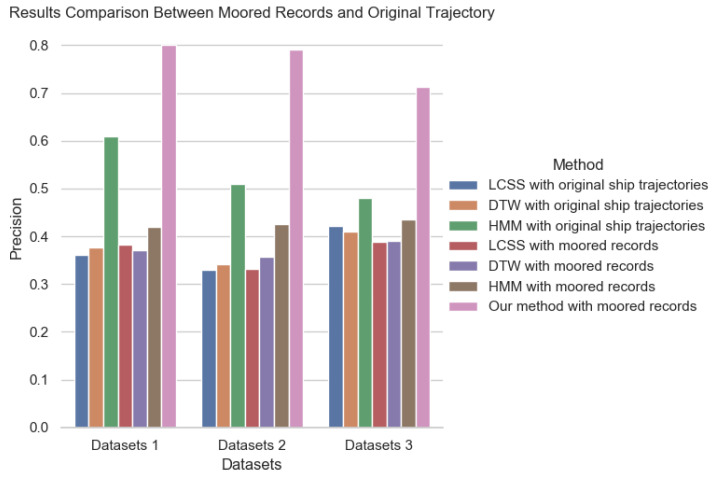
Results comparison of ship classification under moored records and original ship trajectories.

In this section, we answer two questions: (1) Does using moored records better classify ships than using origin ship trajectories? (2) Can Hi-STEM classify ships better than other sequence classification methods? At the same time, for the sake of comparing model performance in a different amount of training data, we reduce the training data to three months and one month, keeping the test data invariant concurrently. First, we employ three common trajectory classification methods, including LCSS, DTW, and HMM. Those method can be realized by open source code: https://pypi.org/project/hmmlearn/ (accessed on 25 November 2021); https://github.com/markdregan/K-Nearest-Neighbors-with-Dynamic-Time-Warping (accessed on 25 November 2021). We compare them with our method based on moored records. The results are shown in Figure 5.

We can see from Figure 5 that the ship classification effect based on the original trajectory is much lower than that based on moored records. In particular, the performance of ship classification based on trajectory similarity is very poor. This is because the trajectories of the same type of ships are very different, and it is difficult to capture the movement patterns of the same type of ships by LCSS or DTW. The performance based on HMM is better than that based on trajectory similarity measurement. However, due to the large uncertainty in ship movement, the HMM method still cannot achieve a good effect with limited data. In addition, we can draw the conclusion that whether in the original trajectory or in the moored records, the effect of the method based on LCSS, DTWm and HMM are not satisfactory. This is because both LCSS and DTW measure the distance between trajectories, but the trajectories of ships of the same type are not close to each other. Our method is to identify the transfer patterns of ships between different ports to classify ships. Because the transfer patterns of different types of ships between ports are very different, our method can achieve a better effect. On the other hand, Hi-STEM is designed for classify moored record, as a result, it is not suitable for original trajectories. We can also see that there is little difference between LCSS and DTW under the original trajectory and moored records, because the difficulty of trajectory-based ship classification is to identify the pattern of ship movement. However, LCSS and DTW only consider the distance between trajectories, and the effect of ship classification is similar under the original trajectories and moored records trajectories. Hi-STEM considers the semantics of each port, and captures the transfer mode of ships between different ports through deep neural network to infer the ship type, which will bring great improvement.

**Figure 6:**
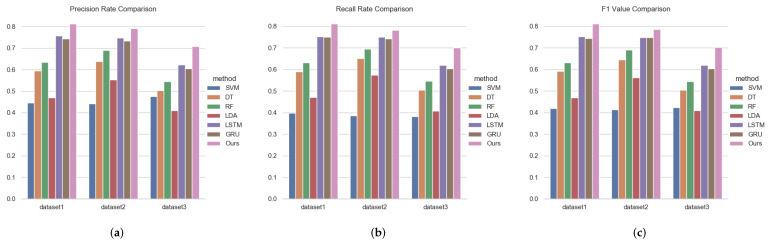
Overall result comparison. The three figure, respectively, show the comparison of ship classification effects under different metric methods. Different colors in each figure represent different methods. (**a**) Precision Rate; (**b**) Recall Rate; (**c**) F1 Value.

On the other hand, we compare Hi-STEM with other sequence classification method based on moored records. The overall results are shown in Figure 6.

As we can see from Figure 6, we obtain the following conclusions: (1) Trajectory classification results on SVM, DT, RF are inferior. This is because real trajectory data are highly free on movement space, making it challenging to find the classification plane on movement space. (2) Hi-STEM is better than the RNN-based model. Compared with the RNN-based model, Hi-STEM has two improvements. Hi-STEM learned the semantic representation of spatial and temporal from historical trajectory data. Hi-STEM introduces the attention mechanism and considers the influence of different locations on the classification results. (3) We can also see that with the decrease of the number of training samples (historical data), all methods’ accuracy shows a downward trend, but Hi-STEM decreases slowly than existing methods. However, when the training data drops to three months, the result accuracy of the RNN-based method approach very rapidly, but our method’s accuracy remains the same. When the training data is down to only one month, all methods’ results are significantly reduced, but Hi-STEM still shows better accuracy than others. There are two reasons we think Hi-STEM outperforms other methods when the number of training samples decreases: (1) Hi-STEM introduces information in space embedding and time embedding, which is independent of training data and will not decrease with the reduction of training data. (2) Hi stem introduces the attention mechanism, which can more focus on capturing different types of features in the trajectory, which will reduce the dependence on the amount of training data.

### 3.4. Robustness Analysis

**Figure 7:**
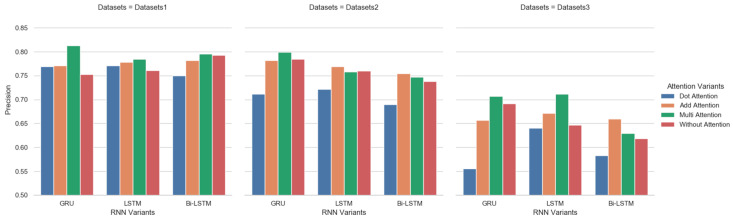
Result comparison with different RNNs units and attention scores. The figure describes the influence of different attention mechanisms and RNN models in Hi-STEM on the results under different data sets.

In this section, we verify the robustness of our method. We first analyze Hi-STEM performance under different parameters. We compare Hi-STEM results under different RNN units and different attention score measures. We choose LSTM, GRU, and BRNN as RNN units’ variants and additive, multiplicative, and dot attention scores. By combining different RNN variants and attention scores, we can obtain nine kinds of Hi-STEM variants. We conduct experiments on those nine kinds of Hi-STEM variants with three datasets. The results are shown in Figure 7.

From Figure 7, we can see that Hi-STEM with GRU units performs worse than other RNN units. Hi-STEM performance with LSTM units is relatively stable, and results are at the same level under different attention scores. Add attention are better than the other two attention scores. We can draw the conclusion that (1) Attention mechanism did affects classification results. In most situations, the attention mechanism is able to improve the accuracy of ship classification. (2) When the training data is sufficient, there is little difference in the impact of different attention calculation methods on the results. However, when training data reduced, different attention mechanisms will fluctuate greatly.

**Figure 8:**
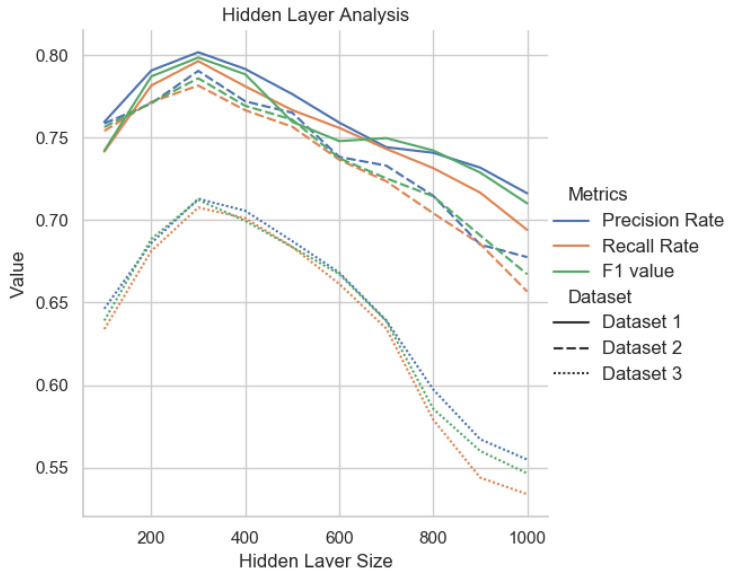
Hi-STEM results with different hidden layer length. Different colors represent different indicators, and different lines represent different data sets. It can be seen from the figure that under different data sets, the effect of the model is the best when the hidden layer size is 300.

Second, we compare the effect of hidden layer size on trajectory classification accuracy. We select the hidden layer size from 100 to 1000 and compare the results under different hidden layers as shown in Figure 8. We can see that when the hidden layer size is less than 300, the accuracy of the model will increase with the increase of the hidden layer size. However, the classification accuracy of Hi-STEM does not change significantly with the setting of other hidden layer sizes, which indicates that Hi-STEM is not sensitive to this parameter.

### 3.5. Embedding Visualization Analysis

**Figure 9:**
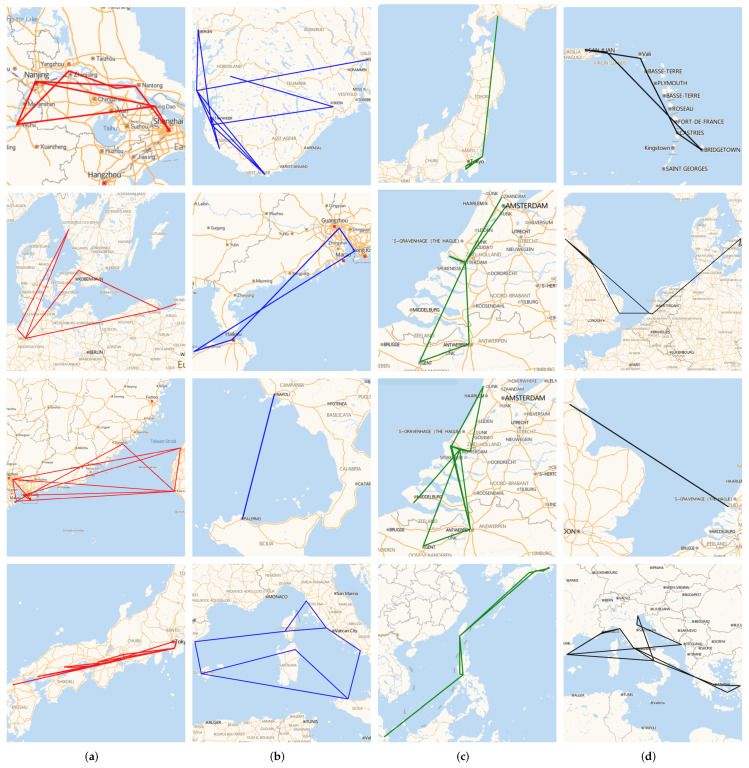
Ship moored records examples. We show the moored records of four different types of ships. The moored records of four ships in a month are selected randomly from each type of ship for visualization. Each column in the map represents the moored records of ships of the same kind, and the colors of their moored records are also the same on the map. We can find that the same kind of ship’s moored records has no apparent similarity in both the geographical spatial distribution and the motion pattern. (**a**) Cargo; (**b**) Fishing; (**c**) Oil; (**d**) Passenger.

Finally, we want to explore how Hi-STEM works. Because the working principle of the internal structure of the deep neural network is still a black box for us, we visualize the input and output of the neural network. For the input end, first, we randomly select four ships from four different ship classifications, and extract their trajectories for one month and visualize them on the map, which is shown in Figure 9

**Figure 10:**
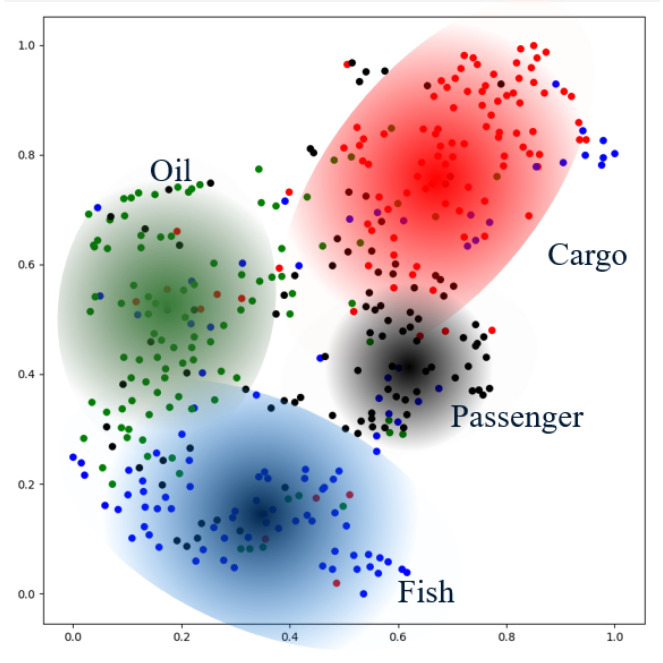
Ship movement embedding vectors visualization. The points shown in the figure are the high-dimensional vector points in the two-dimensional space after dimension reduction. These points are one-to-one corresponding to the original space’s ship movement, and the points with different colors represent different types of ships in the original space. We described the cluster and marked the specific the type of ship next to the cluster.

Then for the output end, we visualize the latent vector of moored records. Because embedded moored records are still high-dimensional vectors, we employ t-distributed Stochastic Neighbor Embedding, and t-SNE [33,34] for embedded moored records dimensionality reduction. It is a nonlinear dimensionality reduction technique well-suited for embedding high-dimensional data for visualization in a low-dimensional space of two or three dimensions. More precisely stated, it models each high-dimensional object by a two- or three-dimensional point in such a way that similar objects are modeled by nearby points, and dissimilar objects are modeled by distant points with high probability. In practice, we use the public machine learning tools (https://scikit-learn.org/stable/modules/generated/sklearn.manifold.TSNE.html, accessed on 25 November 2021) to realize the t-SNE method. The visualized embedded vectors are shown as Figure 10.

As we can see, the movement of the same type of ship in the original space has a very low correlation. It is difficult to find similar features in the same ship movement pattern. However, in the embedding space, the same type of ships is clustered in near areas. Hi-STEM can project the same kind of trajectory to the adjacent points in the feature space, which makes it available to find the effective classification plane in the feature space.

## 4. Conclusions

This paper reviewed the problem of ship type classification. To address the challenges of high uncertainty of real trajectories, we proposed a hierarchical spatial-temporal embedding method based on enhanced trajectory features, which can map origin trajectory into the feature space and find the classification plane in the feature space. We conducted experiments on the real-world trajectory datasets and the results show that our method has high accuracy. Furthermore, as the size of training data reduces, the model performs much better than other approaches. We compared the performance under different parameters to test the robustness of the method. Attention mechanism was also introduced into our method which makes the model more accurate. In addition, we visualized embedded vectors and observed the same type of ship clustering in the feature space. In future works, we will work out ship classification datasets that can be used by more scholars and make them available to researchers in related fields.

## Figures and Tables

**Table 1:** Training data description and statistics.

Attribute	N	T	Avr	TR
Datasets				
Training Dataset 1	400	1200	16.9	2015.05∼2015.11
Training Dataset 2	400	800	13.6	2015.05∼2015.08
Training Dataset 3	400	400	14.5	2015.05∼2015.06
Test Data	400	400	15.53	2015.04∼2015.05

*N* and *T* in the above table represents the number of ships and the number of trajectory in the dataset. *Avr* is the average number of locations per trajectory. *TR* represents the collection time range of the dataset.

## Data Availability

The data presented in this study are openly available at https://github.com/taos123/Ship_Classification_Moored (accessed on 25 November 2021).
